# CT Image Reconstruction from Sparse Projections Using Adaptive TpV Regularization

**DOI:** 10.1155/2015/354869

**Published:** 2015-05-18

**Authors:** Hongliang Qi, Zijia Chen, Linghong Zhou

**Affiliations:** School of Biomedical Engineering, Southern Medical University, Guangzhou 510515, China

## Abstract

Radiation dose reduction without losing CT image quality has been an increasing concern. Reducing the number of X-ray projections to reconstruct CT images, which is also called sparse-projection reconstruction, can potentially avoid excessive dose delivered to patients in CT examination. To overcome the disadvantages of total variation (TV) minimization method, in this work we introduce a novel adaptive TpV regularization into sparse-projection image reconstruction and use FISTA technique to accelerate iterative convergence. The numerical experiments demonstrate that the proposed method suppresses noise and artifacts more efficiently, and preserves structure information better than other existing reconstruction methods.

## 1. Introduction

X-ray computed tomography (CT), as an important medical imaging protocol, has been widely used in clinical applications. However, the involved X-ray radiation dose delivered to patients may potentially increase the probability of causing cancer [1–4]. In this sense, reducing radiation dose without significantly losing image quality is highly required.

Radiation dose in CT examination can be reduced by decreasing the number of projections. However, conventional filtered back-projection (FBP) reconstruction algorithm suffers from systematic geometric distortion and streak artifacts when the measured projection data is not sufficient [5–7]. Iterative methods have been proposed to overcome this problem. Recently, compressed sensing (CS) theory [8] has been applied in CT image reconstruction. It is possible to reconstruct high-quality images from sparse-projection data under the frame of CS. Many optimization methods have been studied following such concepts. Among these optimization methods, total variation (TV) minimization has been widely used. The most famous reconstruction model with TV is ART-TV, firstly proposed by Sidky et al. [9, 10]. This method consists of two steps: ART reconstruction and TV minimization. However, TV is based on an assumption that the signal is piecewise smooth, so this makes TV algorithm suffer from oversmoothing in image edges. To solve this problem, many improved TV methods have been proposed. Tian et al. proposed a TV-based edge preserving (EPTV) model [11]. This model can preserve edges by bringing in different weights in the TV term from edges and constant areas of the to-be-estimated image. Different from the EPTV model, Liu et al. considered the anisotropic edge property of an image and proposed a novel adaptive-weighted TV (AwTV) model [12] for low-dose CT image reconstruction from sparse-sampled projection data. Zhang et al. used a high-order norm coupled within TV to overcome the disadvantages of traditional TV minimization [13]. Chang et al. proposed a few-view reweighed sparsity hunting (FRESH) method for CT image reconstruction [14]. Sidky et al. replaced *L*_1_ norm with *L*_*p*_  (0 < *p* < 1) norm in the minimization function and investigated image reconstruction by minimizing the *L*_*p*_ norm of the image gradient magnitude or the so-called total *p*-variation (TpV) [15]. Chen et al. proposed a CT reconstruction algorithm based on *L*_*p*_  (*p* = 1/2) regularization, where *L*_1/2_ norm is used as the regularization norm and gradient as the sparse conversion [16]. However, the TpV and *L*_*p*_  (*p* = 1/2) regularization methods choose *p* value as a constant in the whole image without identifying edges and constant areas. The disadvantage is that larger *p* value can oversmooth edges and sometimes produce blocky artifacts, while smaller *p* value can preserve edges well but enhance blocky artifacts in constant areas when the projection data is noisy (as shown in Figure 3(f) in [16]). The blocky artifacts are introduced by the noise in the projections whenever *p* is less than 1 or *p* = 1. Although *L*_0_ regularization is the sparest and most ideal regularization norm, *L*_0_-norm minimization problem is known to be NP-hard, and it is difficult to solve equations. Theoretically, a regularization, which is closer to *L*_0_ norm, could obtain higher-quality CT images in CT reconstruction. It should be noted that TV is the *L*_1_ norm of gradient image. Traditional TpV (0 < *p* < 1) is sparser than TV, and the success of traditional TpV is sharpening image edges, but blocky artifacts still exist in homogeneous regions due to the noisy projection data. The same disadvantage of TV and TpV is their tendency to uniformly penalize the image gradient irrespective of the underlying image structures.

In this study, to deal with the trade-off between smoothing nonedge part and preserving edge part of the image, we propose a CT reconstruction algorithm using adaptive TpV regularization wherein each pixel in reconstructed image corresponds to one *p* value determined by the pixel's gradient magnitude. From our experiments, one can see that the low-contrast features can be reconstructed better than other methods and blocky artifacts are reduced much to a certain extent. The rest of the paper is organized as follows. In Section 2, ART-TV, traditional TpV, adaptive TpV regularization, and the proposed CT reconstruction algorithm are introduced, respectively. In Section 3, quantitative and qualitative experimental results are shown.  Section 4 concludes the paper.

## 2. Materials and Methods

### 2.1. ART-TV Reconstruction

CT reconstruction problem can be converted to a constrained optimization problem

(1) $$\begin{array}{c} f=\underset{f}{\arg\;\min}\;R\left(f\right)\;\text{subject}\;\text{to}\;Af=y, \end{array}$$

where *A* = {*a*_*ij*_} denotes the system matrix, *y* represents the projection data, and *f* is the reconstructed image. *R*(*f*) is the regularization function.

To solve 1, Sidky et al. proposed famous total variation (TV) based reconstruction method (ART-TV). In their method, *R*(*f*) in 1 was considered as a *L*_1_ norm of the first-order gradient image or the so-called TV norm. In a 2D image *f* with the size *L* × *M*, whose pixel values are labeled by *f*_*l*,*m*_, its gradient magnitude with respect to *f*_*l*,*m*_ can be expressed as

(2) ∇fl,m=fl,m−fl−1,m2+fl,m−fl,m−12,     ihhh2≤l≤L, 2≤m≤M.

The TV of image *f* is defined as

(3) fTV=∑l,mL,M∇fl,m=∑l,mL,Mfl,m−fl−1,m2+fl,m−fl,m−12+ε,

where the parameter *ε* is a small positive constant to avoid discontinuities.

The ART-TV method is implemented by performing ART algorithm as the first step and TV minimization using gradient descent method as the second step. One can see [9] for more implementation details.

### 2.2. Traditional TpV and Adaptive TpV Regularization

For traditional TpV algorithm, the quantity ‖∇*f*‖_*p*_ is the *p*-norm of the image gradient magnitude. For a 2D image it can be defined by

(4) fTpV=∑l,mL,M∇fl,mp=∑l,mL,M(fl,m−fl−1,m)2+(fl,m−fl,m−1)2+εp,hhhhhhhhhhhhhhhhhhhhhhhhhhhh0<p≤1.

When *p* is set to be 1, the ‖*f*‖_TpV_ reduces to conventional ‖*f*‖_TV_ and reconstructed image will suffer from oversmoothing image feature details. When *p* < 1 (e.g., *p* = 1/2 in [16]), the structural information can be efficiently preserved, but at the same time the blocky artifacts in nonedge regions will be enhanced when the projection data is noisy.

To overcome this limitation, in this work we propose an adaptive TpV (ATpV) regularization defined by

(5) fATpV =∑l,mL,M(fl,m−fl−1,m)2+(fl,m−fl,m−1)2+εpl,m,hhhhhhhhhhhhhhhhhhhhhhhhhhhhh0<pl,m≤1,

where *p*_*l*,*m*_ is determined by pixel *f*_*l*,*m*_ in a 2D image. On one hand, if a pixel's gradient magnitude is large, this pixel is on the edge and corresponds to a small *p* value to avoid oversmoothing edges. On the other hand, if the gradient magnitude of one pixel is small, this pixel is in the nonedge area and corresponds to a large *p* value to suppress noise and artifacts. In this study, we define *p*_*l*,*m*_ as

(6) $$\begin{array}{c} p_{l,m}=\frac{1}{1+\nabla {\left(B*f\right)}_{l,m}}, \end{array}$$

where *B*∗*f* denotes image *f* is filtered by the well-known bilateral filter which does well in denoising and preserving edge information. ∇ is gradient operator. Adding bilateral filter on image *f* is to avoid treating noise point as edge point when calculating gradient magnitude. Obviously in 6, a pixel with large gradient magnitude corresponds to a small *p* value and a pixel with small gradient magnitude corresponds to a large *p* value. The “1” in the denominator in 6 may not be the best option and may be correlated with the contrast value of the reconstruction, but in our experiments, the defined 6 can produce good result. How to replace “1” with an optimal value will be an interesting topic in our future study.

In summary, the benefit of the proposed ATpV is that the parameter *p* is dynamically adopted by identifying edges and nonedges, and larger *p* value is chosen to smooth constant areas while smaller *p* value is chosen to preserve edge part, which will improve the reconstruction quality for sparse-view reconstruction.

### 2.3. CT Reconstruction Algorithm Based on Adaptive TpV Regularization

According to aforementioned methods, in this paper we propose CT image iterative reconstruction using ATpV regularization. The reconstruction is implemented by solving the following constrained minimization problem:

(7) f=arg min⁡f⁡fATpV=arg⁡min⁡f⁡∑l,mL,M(fl,m−fl−1,m)2+(fl,m−fl,m−1)2+εpl,m subject  to  Af=y.

The algorithm implementation can follow ART-TV in [9].

Besides, we apply fast iterative shrinkage/thresholding algorithm (FISTA) [17] to accelerate iterative convergence. In FISTA, the key idea is that initial value of the next iteration is determined by a linear combination of the two previous iterate results. For simplicity, in this study the proposed reconstruction method is termed ART-ATpV without using FISTA or ART-ATpV-FISTA using FISTA.

In summary, the main steps of ART-ATpV-FISTA are as follows.

(A) Initialization: *f* ^0^ = 0, iteration index *k* = 1, 2 … *K*, *t*_0_ = 0.
(B) ART reconstruction:
  (8) $$\begin{array}{c} f^{k+1}=f^{k}+\lambda A_{i}\frac{y_{i}-A_{i}f^{k}}{A_{i}A_{i}^{T}}. \end{array}$$
(C) Positivity constraint: *f* ^*k*+1^ = max⁡(*f* ^*k*+1^, 0).
(D) ATpV minimization:

 calculate *p*_*j*_ = 1/(1 + ∇(*B*∗*f* ^*k*+1^)_*j*_) for each pixel *f*_*j*_^*k*+1^,
 minimize ‖*f*‖_ATpV_ using gradient descent algorithm to get updated *f*_*j*_^*k*+1^.
(E) FISTA acceleration:
  (9) $$\begin{array}{c} t=\frac{1+\sqrt{1+4t_{0}^{2}}}{2},\\ f_{j}^{k+1}=f_{j}^{{\;}^{k+1}}+\frac{t_{0}-1}{t}\left(f_{j}^{{\;}^{k+1}}-f_{j}^{k}\right),\\ t_{0}=t,\;k=k+1. \end{array}$$
(F) Return to (B) until the stopping criterion is satisfied.

In our experimental implementation, the initial to-be-reconstructed image was set to be uniform with pixel values of 0. The relaxation parameter *λ* for the ART was fixed at 1.0, and the step-size used in ATpV minimization using the gradient descent was set to be constant 0.2. The parameter *ε* in 7 was fixed as 10^−5^.

## 3. Experimental Study

### 3.1. Numerical Simulation

**Figure 1:**
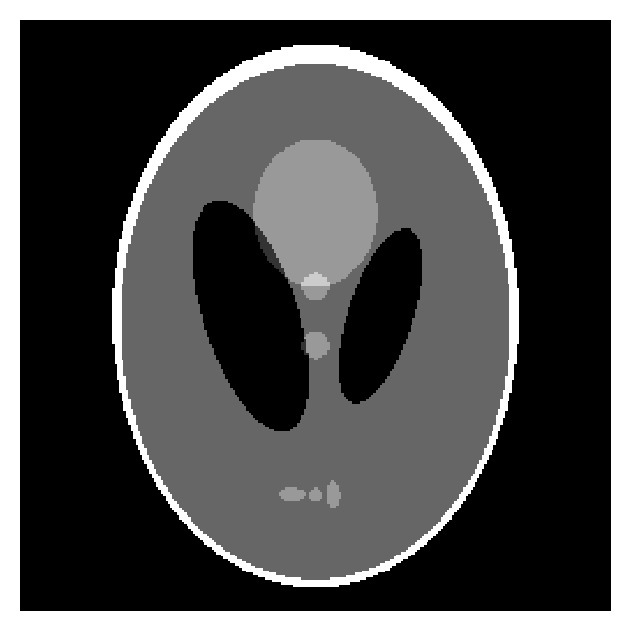
Shepp-Logan phantom.

**Figure 2:**
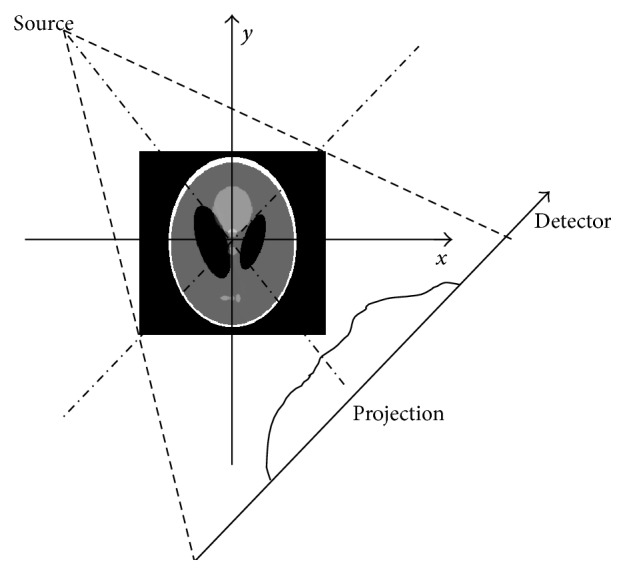
Fan beam CT geometry configuration.

In this section, we study the ART, ART-TV, ART-TpV (*p* = 0.5), and our proposed algorithm. Numerical experiment results are given. Shepp-Logan phantom is tested in this paper as shown in Figure 1, and the size of phantom image is 256 × 256. Without losing generality, we choose a fan beam imaging geometry to capture the projection data as illustrated in Figure 2. The source to rotation center distance is 40 cm and the detector to rotation center is 40 cm. The image array is 25.6 × 25.6 cm^2^. The detector whose length is 61.44 cm is modeled as a straight-line array of 512 detector bins. All the tests are performed by MATLAB on a PC with Intel (R) Core (TM) 2 Quad CPU 2.50 GHz and 3.25 GB RAM.

The generation of projection data is using Siddon's ray-driven algorithm [18, 19]. Projection data is sampled evenly over 360° and the number of projections in this experiment is set 20 over 360°. In the simulation, we add 0.2% Gaussian noise to noise-free projection data. The iteration number for all iterative methods in this experiment is 200, which makes sure each method reaches convergence.

**Figure 3:**
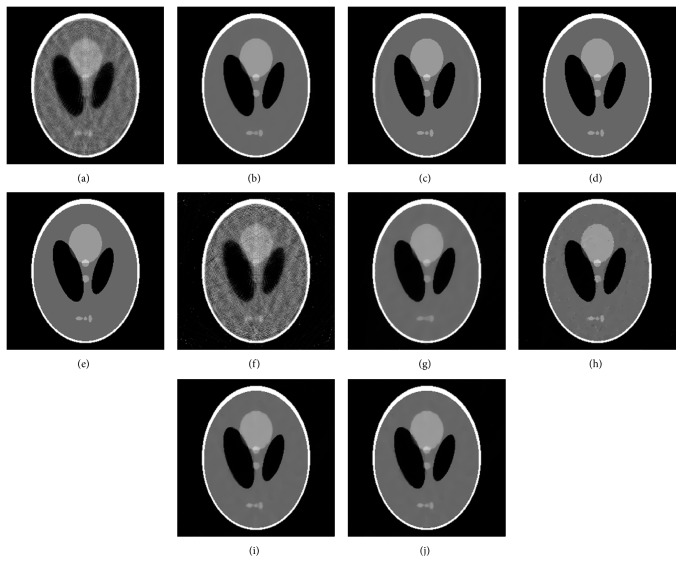
The images reconstructed by different reconstruction algorithms from the noise-free and noisy data. ((a)–(e)) Reconstructed images from noise-free data: (a) image reconstructed by ART, (b) image reconstructed by ART-TV, (c) image reconstructed by ART-TpV, (d) image reconstructed by ART-ATpV, and (e) image reconstructed by ART-ATpV-FISTA; ((f)–(j)) reconstructed images from noisy data: (f) image reconstructed by ART, (g) image reconstructed by ART-TV, (h) image reconstructed by ART-TpV, (i) image reconstructed by ART-ATpV, and (j) image reconstructed by ART-ATpV-FISTA.

The reconstruction results are shown in Figure 3. Figures 3(a)–3(e) show the reconstructed images using different methods from noise-free projection data. In Figure 3(a), it is obvious that, due to insufficiency of projection data, ART reconstructs low-quality image containing severe artifacts and noises. In Figure 3(b), artifacts and noises are effectively suppressed by using ART-TV, but three oval organs in the bottom of image are blurred to some extent. The ART-TpV can overcome the limitation of TV and the result can be seen from Figure 3(c). Similarly, the image edges reconstructed by ART-ATpV are well kept and three oval organs in the bottom of image are more distinguishable in Figure 3(d).  Figure 3(e) shows the image reconstructed by ART-ATpV-FISTA after only 50 iterations which has almost the same image quality as Figure 3(d). One can see that ART-ATpV-FISTA can reconstruct high-quality image using less iterations. The same results can be seen in Figures 3(f)–3(j) with noisy projection data. It is noticed that image (Figure 3(h)) reconstructed by ART-TpV has some small enhanced blocky artifacts due to noise existing in projection data.

**Figure 4:**
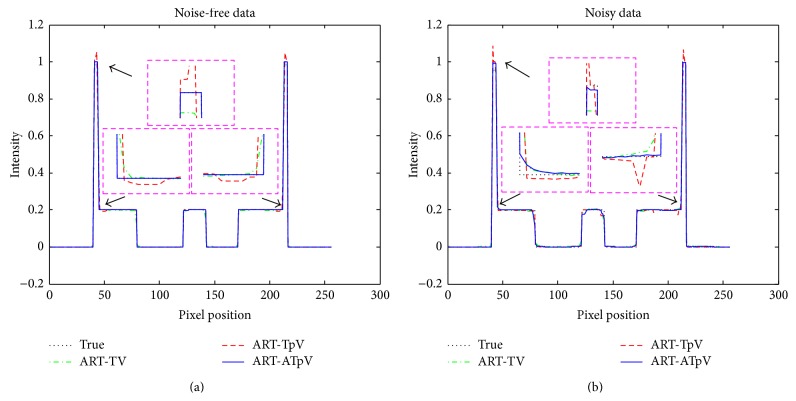
The comparison between reconstructed images using three different reconstruction algorithms and original Shepp-Logan phantom. (a) The horizontal profiles in reconstructed images using ART-TV, ART-TpV, and ART-ATpV methods from noise-free data and original Shepp-Logan phantom and (b) the horizontal profiles in reconstructed images using ART-TV, ART-TpV, and ART-ATpV methods from noisy data and original Shepp-Logan image.

Figure 4 depicts the horizontal profiles through the center pixels of the ART-TV, ART-TpV, and ART-ATpV reconstructed images corresponding to Figure 3. It can be clearly seen that the ART-ATpV algorithm produces a closer profile to the true image compared to ART-TV and ART-TpV.

To assess the accuracy of the reconstructed image, the mean absolute error (MAE) is used and defined by

(10) $$\begin{array}{c} \text{MAE}=\frac{{\left\|x^{}-x^{\text{true}}\right\|}_{1}}{N}, \end{array}$$

where *N* is the total number of pixels of the reconstructed image *x* and *x* ^true^ is the original image.

**Figure 5:**
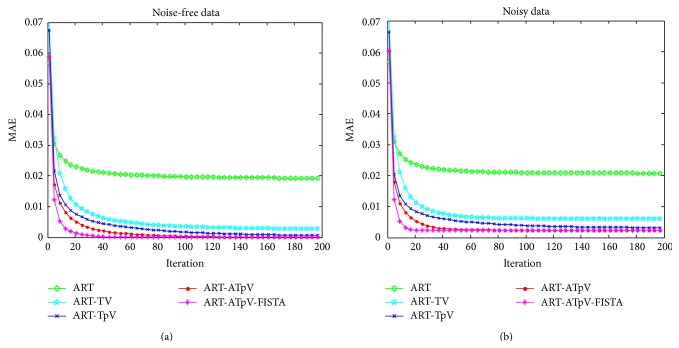
The MAE curves of reconstructed images with different reconstruction algorithms at 20 projection angles and different iteration numbers, and the iteration numbers range from 1 to 200. (a) The MAE curves of reconstructed images from noise-free projection data and (b) the curves of reconstructed images from noisy projection data.

As shown in Figure 5, the ART-ATpV with/without FISTA algorithms can reconstruct high-quality images at less iteration numbers, and application of FISTA to ART-ATpV (denoted ART-ATpV-FISTA) can further remarkably accelerate iterative convergence.

**Figure 6:**
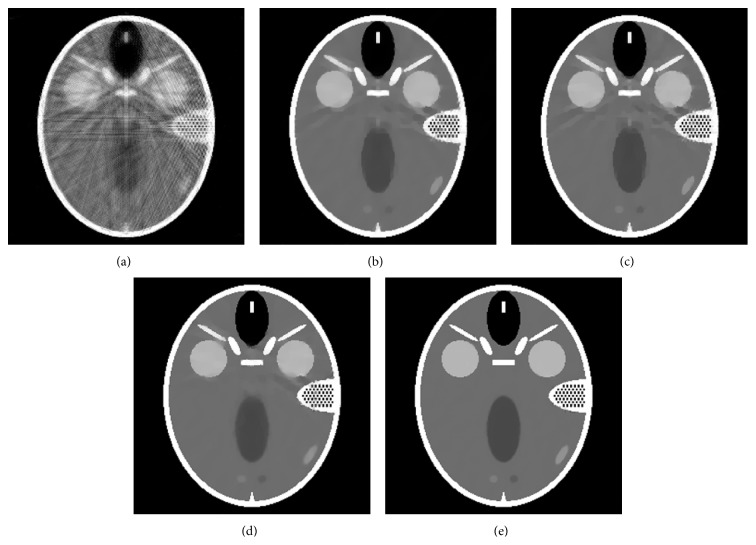
The images reconstructed by different reconstruction algorithms from noisy data: (a) image reconstructed by ART, (b) image reconstructed by ART-TV, (c) image reconstructed by ART-TpV, (d) image reconstructed by ART-ATpV, and (e) image reconstructed by ART-ATpV-FISTA.

To challenge our ART-ATpV/ART-ATpV-FISTA method further, we use a complicated low-contrast FORBILD phantom to reconstruct image and compare it to other methods. The corresponding images are in Figure 6. One can see that high-quality images can be obtained by our proposed method.

### 3.2. Real Data Experiment

In this section, we use a real CT image (head phantom) obtained from a commercial medical CT scanner to test the effectiveness of our ART-ATpV and ART-ATpV-FISTA algorithms and compare them with other algorithms. 40 projections are simulated in this case, with the aforementioned geometrical parameters unchanged. For all iterative methods except ART-ATpV-FISTA, the number of iterations is 50. For ART-ATpV-FISTA, the number of iterations is 20.

**Figure 7:**
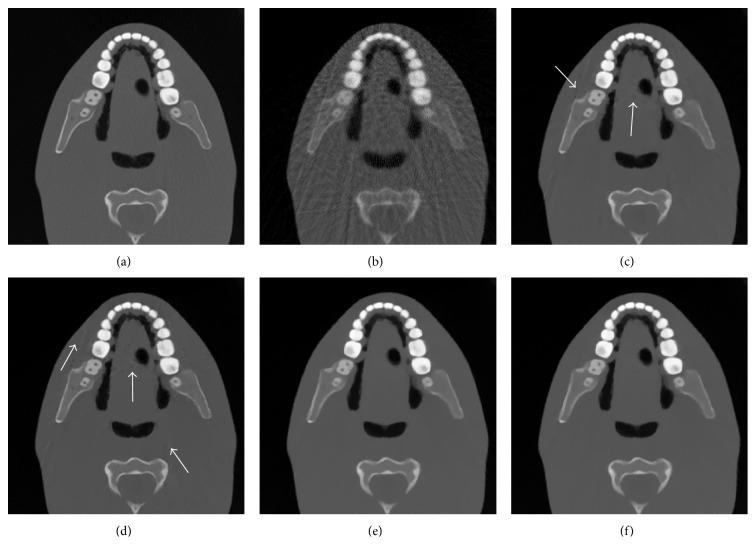
The reconstructed images by different algorithms from real head phantom projection data. (a) The original image, (b) the reconstructed image using ART, (c) the reconstructed image using ART-TV, (d) the reconstructed image using ART-TpV, (e) the reconstructed image using ART-ATpV, and (f) the reconstructed image using ART-ATpV-FISTA.

As shown in Figure 7, due to the incompleteness of projection data, the reconstructed image using ART method has more artifacts and noises than the reconstructed images by using ART-TV, ART-TpV, ART-ATpV, and ART-ATpV-FISTA method. ART-TV suppresses most of the artifacts and noises, but the oversmoothing effect and blocky artifacts exist in the images, as indicated by the white arrows in Figure 7(c). Although ART-TpV method suppresses most of the artifacts and noises, the blocky artifacts are still visible and enhanced in the images, as indicated by the white arrows in Figure 7(d). ART-ATpV overcomes the flaws and preserves the structure information well, without obvious blocky artifacts.  Figure 7(f) shows the image reconstructed by ART-ATpV-FISTA after only 20 iterations. It is seen that the reconstructed image has almost the same image quality as Figure 7(e) (ART-ATpV), which means that ART-ATpV-FISTA can reconstruct high-quality image using less iterations. The FISTA technique is a useful tool to accelerate convergence rate and can be applied to other iterative reconstruction approaches for speedup.

## 4. Discussions and Conclusion

In this paper, we present ART-ATpV-FISTA method for X-ray CT reconstruction from few-view or sparse projections. The main contribution of this work is to minimize adaptive TpV norm of reconstructed image instead of traditional TpV norm and TV norm. FISTA technique is employed to speed up iterative convergence rate.

The advantage of adaptive TpV is that if a pixel's gradient magnitude is large, this pixel is on the edge and corresponds to a small *p* value to avoid oversmoothing. If gradient magnitude of one pixel is small, this pixel is in the constant area and corresponds to a large *p* value to smooth noise and artifacts. In our primary experiment, the Gaussian filter which was chosen in 6 may not lead to some losses of details, because the Shepp-Logan phantom is simple. When we use low-contrast FORBILD phantom to reconstruct images, as we expect, the Gaussian filter causes the losses of low-contrast details in FORBILD phantom. Therefore, to effectively remove noise and preserve low-contrast structures, we use bilateral filter instead of Gaussian filter when we calculate *p* values, and the results are better.

The performance of the propose method is compared to ART, ART-TV, and ART-TpV methods on Shepp-Logan phantom, low-contrast FORBILD phantom, and a real head phantom. Both qualitative and quantitative comparisons are performed to show the proposed method provides more superior results than other existing methods. Since the main goal of this work is to demonstrate the effectiveness of the proposed ATpV-based regularization, the parameters were empirically set through extensive experiments by visual inspection and quantitative measures in this study.

Although the presented ART-ATpV-FISTA algorithm in this paper is used in fan beam CT geometry, it is also easily extended to cone beam CT (CBCT) geometry due to its iterative-correction property. Furthermore, the ART-ATpV-FISTA algorithm may also be useful for other tomographic imaging modalities. In the further research, we will apply the developed algorithm in CBCT system and study the few-view CBCT reconstruction, which will reduce radiation dose as much as possible.

Similar to FBP, serious streaking artifacts in the reconstructed CT images using FDK type algorithms [20, 21] exist when number of X-ray projections is not sufficient. Besides, using GPU to speed up the computationally intensive tasks of CBCT reconstruction has drawn a lot of attention recently [22–26] and we would like to include it in our future study.

In conclusion, the proposed algorithm using adaptive TpV regularization in this work can reconstruct high-quality images from few-view projections and will have great potential clinical applications.
